# Joint-Specific and Cross-Joint Strength Profiles in Relation to Maximal Soccer Kicking Speed

**DOI:** 10.3390/life16040688

**Published:** 2026-04-18

**Authors:** İbrahim Orkun Akcan, Sultan Şenyurt, Tolga Altuğ, Betül Ateş, Şeyma Tuba Acar, Büşra Yücelsoy, Gizem Kızılörs, Christopher B. Taber, Hamza Küçük, Ahmet Serhat Aydın, Mehmet Söyler, Cengiz Ölmez

**Affiliations:** 1 Faculty of Sports Sciences, Erzincan Binali Yıldırım University, Erzincan 24002, Türkiye; orkun.akcan@erzincan.edu.tr; 2 Institute of Health Sciences, Ordu University, Ordu 52200, Türkiye; sultansenyurt1052@gmail.com (S.Ş.); betul.ates17@icloud.com (B.A.); stuba.acar@gmail.com (Ş.T.A.); yucelsoybusra1@gmail.com (B.Y.); 3 Faculty of Sports Sciences, Agri İbrahim Çeçen University, Agri 04100, Türkiye; taltug@agri.edu.tr; 4 Institute of Graduate Studies, Sinop University, Sinop 57000, Türkiye; gkizilors@sinop.edu.tr; 5 Department of Exercise and Sport Science, Sacred Heart University, Fairfield, CT 06825, USA; taberc@sacredheart.edu; 6 Yasar Dogu Faculty of Sport Sciences, Ondokuz Mayis University, Samsun 55200, Türkiye; 7 Institute of Health Sciences, Gazi University, Ankara 06500, Türkiye; serhataydingazi@gmail.com; 8 Vocational School of Social Sciences, Cankiri Karatekin University, Çankırı 18200, Türkiye; 9 Faculty of Sports Sciences, Ordu University, Ordu 52200, Türkiye; cengizolmez@odu.edu.tr

**Keywords:** ankle strength, ball speed, kinetic chain, soccer kicking, strength profiling

## Abstract

The purpose of this study was to examine associations between lower-limb mechanical strength, phase-oriented composite strength indices, generalized neuromuscular activation, and maximal soccer ball kicking speed in trained athletes. Twenty-five male soccer players (age: 20.64 ± 2.50 years; height: 179.28 ± 4.27 cm; body mass: 75.80 ± 9.41 kg) participated in this cross-sectional study. Isometric ankle and knee joint torques were assessed using an isokinetic dynamometer, and joint-specific and phase-oriented cross-joint composite indices were computed to represent integrated strength capacity across the kinetic chain. Neuromuscular activation was evaluated via surface electromyography during a standardized squat jump task. Ball-kicking speed was measured using Doppler radar during maximal instep kicks. Associations were analyzed using Pearson correlation coefficients (*p* ≤ 0.05) with false discovery rate correction for multiple comparisons. In unadjusted analyses, moderate positive correlations were observed for several ankle torque variables and composite ankle strength indices, while swing-phase composite measures demonstrated moderate correlations (r = 0.43–0.55). Knee strength indices and sEMG variables showed no significant relationships. However, none of the variables remained statistically significant after FDR correction, suggesting limited independent explanatory value of isolated isometric strength and non-task-specific neuromuscular activation assessed during a standardized squat jump for maximal kicking performance.

## 1. Introduction

Soccer kicking performance is a complex motor skill that depends on the coordinated interaction of mechanical and neuromuscular factors across the lower-limb kinetic chain [1,2,3]. From a biomechanical perspective, maximal ball speed during instep kicking is commonly described within a proximal-to-distal sequencing framework, whereby mechanical energy generated by proximal segments is transferred and amplified through distal segments prior to ball contact [4,5,6]. Within this framework, effective ball speed generation arises from the coordinated timing of segmental rotations, intersegmental coupling, and joint-level dynamics rather than from the isolated contributions of a single joint or muscle group [2,7,8].

Previous research has emphasized knee extensor strength due to its role in leg swing acceleration and its association with lower-limb power production [9,10,11]. Accordingly, several studies have focused on knee extensor and flexor strength capacities as primary mechanical determinants of kicking performance [9,10,11]. However, findings remain inconsistent, suggesting that isolated measures of knee torque may have limited explanatory value for inter-individual differences in maximal kicking performance [4,12]. Isolated joint torque measurements obtained under static or quasi-static conditions may not fully capture the functional demands of high-speed ballistic skills such as soccer kicking. In particular, single-direction torque outputs provide limited information regarding intermuscular coordination, joint stabilization, and the capacity to regulate stiffness during impact, all of which are critical for effective force transfer at ball contact [1,4,13]. Consistent with this view, recent evidence suggests that maximal torque magnitude alone may not sufficiently explain inter-individual variability in kicking ball speed [14,15].

Other investigations have examined the functional role of the ankle joint in kicking performance [1,4]. The ankle plays a crucial role in controlling foot orientation, modulating joint stiffness, and facilitating efficient energy transmission to the ball during the final phase of the kicking motion [1,4,9]. Importantly, ankle function during kicking is governed not only by the magnitude of plantar flexion or dorsiflexion torque in isolation but also by the integrated action of agonist and antagonist muscle groups that collectively contribute to joint stability and impact control [4,7]. Accordingly, ankle strength capacity may be more appropriately conceptualized as a joint-level property reflecting integrated force generation and stabilization demands rather than as isolated torque production in a single movement direction.

Despite its theoretical relevance, ankle strength has often been treated as a secondary contributor, and few studies have incorporated composite indices integrating multiple ankle strength components to represent functional joint capacity [1,9]. Composite strength indices may offer a more ecologically valid representation of joint behavior by accounting for the combined contributions of opposing muscle actions and by reducing the dimensionality of correlated strength variables [2,4]. Recent evidence linking plantar flexor strength to high-intensity soccer actions [16] and sprint acceleration performance [17] further supports the functional relevance of ankle strength capacity for explosive lower-limb actions.

From a neuromuscular perspective, surface electromyography (sEMG) has been widely used to examine muscle activation patterns during soccer-specific and non-specific tasks [18,19,20]. While generalized neuromuscular activation assessed during standardized explosive movements such as squat jumps provides insight into overall motor output capacity, such measures may be insufficiently sensitive to explain performance variability in highly task-specific skills requiring precise temporal coordination [18,19]. In these contexts, EMG amplitude alone may not adequately reflect the timing, coordination, and intermuscular interactions required for efficient kinetic chain function [18,20,21].

To date, while related approaches have been explored in previous research, direct comparisons of composite ankle strength, knee strength, and generalized neuromuscular activation within the same cohort remain limited in the context of maximal soccer kicking performance [1,9,20]. Soccer kicking is a highly dynamic ballistic action characterized by rapid intersegmental coordination and stretch–shortening cycle involvement. Within this context, isolated isometric strength assessments provide controlled estimates of maximal joint-level force-generating capacity, although they do not fully capture the velocity-dependent and coordinative characteristics of the kicking action. This represents a reductionist approach that may not fully reflect the integrated and task-specific nature of soccer kicking performance. Accordingly, the present study adopted this approach to examine whether such isolated strength characteristics are meaningfully associated with maximal kicking performance. Therefore, the purpose of the present study was to examine the associations between composite ankle strength, knee strength, and generalized neuromuscular activation and ball speed during soccer kicking in trained male players. By contrasting composite joint-level strength indices with traditional isolated strength measures and generalized neuromuscular activation, this study aims to provide a more functionally grounded evaluation of the mechanical and neuromuscular factors associated with maximal kicking performance. It was hypothesized that composite strength indices may be more strongly associated with kicking ball speed than isolated joint-level strength measures and generalized neuromuscular activation.

## 2. Materials and Methods

### 2.1. Study Design

The present study employed a cross-sectional design to examine the relative associations between lower-limb mechanical strength, general neuromuscular activation, and soccer ball kicking speed under controlled conditions. The experimental approach was designed to isolate joint-specific strength capacity and generalized neuromuscular output from the complex coordinative and technical demands inherent to the soccer kicking task.

Isometric strength testing was selected to quantify maximal joint-level torque capacity at the ankle and knee with high reliability and low measurement variability under controlled conditions. Isometric assessments provide stable and reproducible measures of maximal torque production that are less influenced by movement coordination, joint angular velocity, or skill-dependent execution than dynamic multi-joint tasks [12]. Furthermore, evidence indicates that isometric and dynamic strength represent partially distinct neuromuscular performance characteristics; therefore, isolating isometric torque capacity allows the evaluation of maximal force-generating potential independent of dynamic task coordination [22].

Neuromuscular activation was assessed during a standardized squat jump task to capture general explosive activation patterns under controlled and reproducible conditions. Squat jumping provides a repeatable ballistic framework for assessing lower-limb neuromuscular output, while EMG recorded during such tasks is less affected by technical execution variability and impact-related artifacts than task-specific kicking movements [15,19]. This approach enabled the assessment of generalized neuromuscular activation capacity rather than skill-dependent activation patterns specific to the kicking task. Accordingly, a standardized squat jump task was used to ensure consistent and comparable neuromuscular measurements across participants. However, this approach may not fully capture task-specific neuromuscular activation patterns associated with soccer kicking.

### 2.2. Participants

Twenty-five male soccer players (age: 20.64 ± 2.50 years; height: 179.28 ± 4.27 cm; body mass: 75.80 ± 9.41 kg; BMI: 23.55 ± 2.42 kg/m^2^; training age: 7.16 ± 1.49 years) voluntarily participated in the study. The athletes had been training regularly, at least twice per week, for at least five years. Inclusion criteria were regular participation in competitive soccer training and matches and the absence of a lower-limb injury in the previous six months. Participants with a history of lower-extremity surgery, current musculoskeletal pain, or neurological disorders affecting movement performance were excluded. From an initial pool of 50 eligible athletes, 25 participants were randomly selected using a web-based randomization tool. The subsampling approach was implemented to ensure controlled testing conditions, minimize fatigue across repeated measurements, and maintain data quality within the constraints of the experimental protocol. We acknowledge that including the full sample could have increased statistical power. The dominant limb was defined as the preferred kicking leg used to perform a maximal instep kick, while the contralateral limb was defined as the non-dominant (support) leg.

The required sample size was determined a priori using G*Power software (version 3.1.9.6, University of Düsseldorf, Düsseldorf, Germany). Assuming a large effect size (f = 0.6), an alpha level of 0.05, and a statistical power of 0.80, the minimum required sample size was calculated as 25 participants [23].

All participants were fully informed about the study objectives, procedures, potential benefits, and risks prior to participation, and written informed consent was obtained in accordance with the Declaration of Helsinki. Ethical approval for the study was granted by the Non-Interventional Sports Science Research Ethics Committee of Ordu University (Approval No: 2025-33).

### 2.3. Procedures

**Figure 1:**
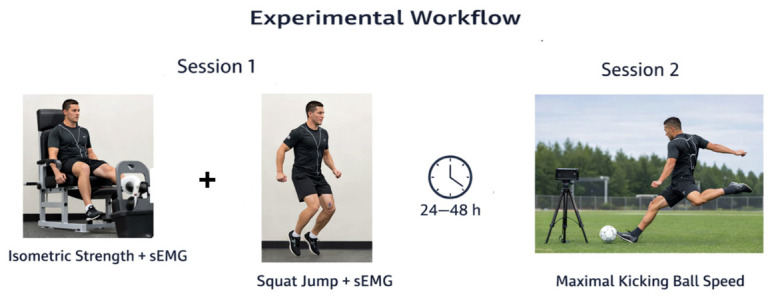
Experimental workflow illustrating the two-session design. In Session 1, isometric strength testing with concurrent sEMG recording and squat jump assessment with sEMG were performed. After a 24–48 h recovery period, Session 2 consisted of maximal instep-kicking trials to assess ball speed.

Testing sessions were conducted under controlled laboratory conditions (21–23 °C) at the same time of day (±1 h) to minimize circadian and environmental variability. Participants were instructed to avoid strenuous exercise, caffeine, and alcohol for 24 h prior to testing and to maintain normal hydration status. The experimental protocol was conducted across two testing sessions to mitigate acute fatigue and ensure independent assessment of strength and performance variables (Figure 1). In the first session, participants’ body mass was measured using a Jawon X-scan Plus II device (Jawon Medical, Seoul, Republic of Korea) while wearing lightweight clothing that did not materially affect the measurement. Session two was dedicated to assessing maximal instep-kicking ball speed using a Bushnell radar gun (model 101911, Bushnell, Overland Park, KS, USA) to minimize fatigue effects from prior testing. Both sessions were performed at the same time of day under standardized pre-test conditions.

### 2.4. Warm-Up and Preparation

Before the warm-up, skin preparation for sEMG recordings was performed to reduce impedance. When necessary, the skin over the target muscles was shaved, lightly abraded, and cleaned with alcohol. After skin preparation was completed, participants performed a standardized warm-up consisting of five minutes of cycling at 60 rpm on a cycle ergometer (COSMED E100, COSMED, Rome, Italy), followed by a dynamic stretching routine targeting the lower limbs. The routine included anterior–posterior and medial–lateral leg swings, walking lunges, high-knee marching, butt-kick drills, and ankle mobility movements. Each exercise was performed for approximately 10–12 repetitions, twice, for a total of about 5 min. Following the warm-up, sEMG electrodes were placed on the prepared skin areas, and all procedures were conducted under the supervision of the same investigator to ensure consistent execution across participants. sEMG signals were recorded using a wireless Noraxon system (Noraxon USA Inc., Scottsdale, AZ, USA). Electrode placement followed SENIAM recommendations [24]. Electrodes were placed on the dominant limb over the following muscles: rectus femoris (RF), soleus (SOL), biceps femoris (BF), tibialis anterior (TA), gastrocnemius medialis (GM), gastrocnemius lateralis (GL), vastus lateralis (VL), and vastus medialis (VM). sEMG recordings were obtained from the dominant limb to provide a standardized representation of neuromuscular activation while reducing methodological complexity.

### 2.5. Isometric Strength Testing and MVIC

Following electrode placement, isometric strength testing was performed using an isokinetic dynamometer (Humac Norm, CSMi, Stoughton, MA, USA). First, sEMG signals were recorded to obtain maximal voluntary isometric contraction (%MVIC) values for EMG normalization. Participants performed 5-s maximal voluntary isometric contractions for knee extension, knee flexion, ankle plantar flexion, and ankle dorsiflexion. For muscle action, three maximal trials were completed, with each trial lasting 5 s. A 60-s rest period was provided between trials, and a 5-min rest interval was allowed between different joint test conditions. Strong verbal encouragement was provided throughout all maximal contractions. Isometric dynamometry and jump-based performance assessments have demonstrated good-to-excellent test–retest reliability (ICC = 0.70–0.94) in athletic populations [25].

### 2.6. Test Positions and Stabilization

Participants were securely strapped at the trunk, pelvis, and tested limb to minimize extraneous movement. The dynamometer rotational axis was carefully aligned with the anatomical joint axis for each test according to standardized isokinetic testing procedures described in the literature and the manufacturer’s recommendations [26,27].

#### 2.6.1. Knee Joint

Knee extension tests were performed in a seated position with the dynamometer chair inclined at approximately 85°, and the knee fixed at 45° of flexion (0° = full extension). Knee flexion tests were performed in the prone position with the knee maintained at the same joint angle. The dynamometer lever arm was attached proximal to the malleoli.

#### 2.6.2. Ankle Joint

Ankle plantar flexion and dorsiflexion tests were conducted in a seated position with the knee flexed at approximately 90° and the ankle fixed in a neutral position (0°). The foot was securely fastened to the footplate adapter.

### 2.7. Torque Variables

For each contraction, peak torque (PT), defined as the highest torque value recorded during the contraction, and average torque (AT), defined as the mean torque over the contraction period, were obtained from the dynamometer software. The highest-performing trial for each variable was retained for analysis. Torque values were normalized to body mass (Nm·kg^−1^). Both peak torque and average torque variables were included in the correlation analyses; however, composite strength indices were derived exclusively from peak torque values.

### 2.8. Squat Jump Assessment

Squat jump performance was assessed concurrently with sEMG recording using a Microgate Witty jump meter (Microgate, Bolzano, Italy), a system that has demonstrated high test–retest reliability for jump height measurements (ICC > 0.90) in athletic populations [28].

Participants performed three maximal squat jumps from a static semi-squat position, without a preparatory countermovement, to ensure a standardized starting condition and minimize the contribution of the stretch–shortening cycle. Before each attempt, participants were instructed to maintain a stable squat position, with the knees at approximately 90° of flexion and the hands fixed on the hips to restrict arm swing. Knee angle standardization was verified using an inertial motion sensor (Noraxon MyoMotion, Scottsdale, AZ, USA) positioned at the mid-thigh of the tested limb, allowing real-time monitoring of joint angle before the jump was initiated. Once the target knee angle was reached and stabilized, participants initiated the jump voluntarily at the moment they felt ready. Two submaximal familiarization jumps were completed before the maximal trials to ensure correct technique and consistent execution across participants. A standardized rest period of 60 s was provided between trials to minimize fatigue. For sEMG analysis, only the concentric propulsion phase of the squat jump (from movement onset to take-off) was considered, while the landing phase was excluded. Jump height was recorded solely to identify the trial associated with the highest performance, and the corresponding sEMG signal from that trial was retained for further analysis. It was not used as an outcome variable, as the primary purpose of the squat jump task was to obtain standardized neuromuscular activation patterns rather than to evaluate explosive performance capacity.

### 2.9. Surface Electromyography (sEMG)

sEMG signals were recorded using a wireless Noraxon system with a sampling frequency of 1000 Hz. Signals were band-pass filtered between 20 and 450 Hz using a fourth-order Butterworth filter, full-wave rectified, and smoothed using a root-mean-square (RMS) algorithm with a 100-ms moving window. These procedures were applied consistently across all muscles and trials.

### 2.10. EMG Normalization

To enable inter-individual and inter-muscle comparisons, sEMG signals were normalized to MVIC values. During the isometric strength tests, sEMG was recorded concurrently with torque measurements. For each muscle, the highest EMG amplitude obtained across all MVIC trials was identified and used as the normalization reference. During the squat jump, sEMG activity was analyzed over the movement phase defined as the period from movement onset to take-off, which was synchronized with the jump height measurement. For each muscle, the mean EMG amplitude within this phase was calculated. Normalized EMG values were then expressed as a percentage of MVIC (%MVIC) according to the following equation:

$$\%MVIC=\frac{EMG_{\mathrm{SJ}}}{EMG_{\mathrm{MVICmax}}}\times 100$$

Values exceeding 100% MVIC may occur due to differences between isometric normalization conditions and dynamic task execution, particularly when dynamic contractions elicit higher activation than that captured during MVIC trials. Therefore, %MVIC values should be interpreted as relative rather than absolute indicators of muscle activation.

### 2.11. Ball Speed Measurement

Ball velocity was measured using a hand-held Doppler radar gun (Bushnell 101911). Radar-based velocity assessments are considered a valid method for measuring movement speed in sport performance settings [29]. In soccer-specific kicking evaluations, behind-goal radar positioning has further demonstrated high concurrent validity and reliability compared with reference radar systems (ICC ≈ 0.95; CCC ≈ 0.99) [30]. Prior to testing, players completed a standardized ball-specific warm-up consisting of five minutes of progressive technical drills, including short passing, controlled dribbling, low-intensity instep kicks, and gradually increasing-intensity shooting movements. This routine was designed to progressively prepare the lower limbs for kicking actions while ensuring consistent technical readiness across participants. The ball-specific warm-up was followed by ten submaximal familiarization penalty kicks from 11 m. Subsequently, participants performed three maximal instep kicks toward a goal (2 m × 3 m) from the same distance. Players were instructed to strike the ball as powerfully as possible while aiming at the center of the goal, which corresponded to the radar measurement axis, to standardize ball trajectory across trials. The radar device was positioned centrally behind the goal along the line of ball trajectory to ensure optimal signal capture. The highest ball velocity was retained for analysis. Ball speed was expressed in kilometers per hour (km·h^−1^).

### 2.12. Statistical Analysis

All statistical analyses were performed using IBM SPSS Statistics for Windows (Version 25.0; IBM Corp., Armonk, NY, USA). Descriptive statistics were calculated for all variables and are presented as mean ± standard deviation (Mean ± SD). The level of statistical significance was set at *p* < 0.05. The normality of data distribution was assessed using the Shapiro–Wilk test and visual inspection of histograms and Q–Q plots. Potential outliers were further examined in relation to measurement error and performance context, and no data points were excluded unless they met both statistical and physiological implausibility criteria. Composite strength indices were constructed using peak torque values to represent integrated joint-level strength capacity.

Joint-specific composite indices were calculated as follows:

- Ankle Strength Index (ASI) = plantar flexion peak torque + dorsiflexion peak torque.
- Knee Strength Index (KSI) = knee extension peak torque + knee flexion peak torque.

In addition to joint-specific indices, phase-oriented composite indices were computed as:

- Push-Phase Strength Index (PPSI) = plantar flexion peak torque + knee extension peak torque.
- Swing-Phase Strength Index (SPSI) = dorsiflexion peak torque + knee flexion peak torque.

The development of these composite indices was conceptually informed by the biomechanical framework of the kinetic chain, which emphasizes the coordinated contribution of multiple joints to force production in ballistic movements. While similar integrative approaches have been used to represent multi-joint function in previous research [1,7,21], the specific configuration of the present indices was designed to reflect joint-specific and phase-oriented contributions within a unified analytical framework. These indices represent simplified constructs rather than direct physiological measures, and their construct validity should be interpreted with caution.

Surface EMG amplitudes recorded during the squat jump task were normalized to maximal voluntary isometric contraction (MVIC) values obtained during the isometric strength assessments and expressed as a percentage of MVIC (%MVIC). The relationships between kicking ball speed and mechanical as well as neuromuscular (EMG) variables were examined using Pearson’s product–moment correlation coefficient (r), as variables satisfied parametric assumptions. Assumptions of linearity and homoscedasticity were verified through visual inspection of scatterplots prior to analysis. Correlation coefficients were interpreted according to conventional thresholds (small: r ≈ 0.10–0.29; moderate: r ≈ 0.30–0.49; large: r ≥ 0.50) [31]. Given the number of correlations tested, false discovery rate (Benjamini–Hochberg) correction was applied to control for type I error [32]. The correction was applied separately to the individual mechanical and neuromuscular variables (Table 1) and to the peak torque-based composite strength indices (Table 2), as these represented distinct hypothesis families. Both unadjusted and adjusted *p* values are reported. Correlation analyses were interpreted as indicative of association rather than causation.

## 3. Results

### 3.1. Individual Mechanical and Neuromuscular Variable

Descriptive statistics for all individual mechanical and neuromuscular variables, together with their associations with kicking ball speed (KBS), are presented in Table 1. All variables met the assumptions of normal distribution based on Shapiro–Wilk tests and visual inspection of histograms and Q–Q plots. Descriptive data are presented as mean ± standard deviation (Mean ± SD) along with minimum–maximum ranges. Isometric strength values differed across joints and movement directions, with knee extension peak torque values exceeding knee flexion and ankle torque values. Dominant ankle plantar flexion peak torque was 61.48 ± 27.93 Nm (18–125), while dominant dorsiflexion peak torque was 57.2 ± 12.95 Nm (41–87).

At the unadjusted level, several ankle torque variables showed moderate unadjusted associations with maximal kicking ball speed. Dominant plantar flexion average torque showed a moderate unadjusted association with KBS (r = 0.41, *p* = 0.04). Dominant dorsiflexion peak torque (r = 0.47, *p* = 0.02) and dominant dorsiflexion peak torque normalized to body mass (r = 0.45, *p* = 0.02) also showed moderate unadjusted associations with KBS. In addition, dominant knee flexion average torque showed a moderate unadjusted association with KBS (r = 0.52, *p* = 0.01). Following application of the Benjamini–Hochberg false discovery rate correction, none of the individual mechanical or neuromuscular variables remained statistically significant (all *p*(FDR) > 0.05). Unadjusted and FDR-adjusted *p* values for all correlations are provided in Table 1.

### 3.2. Composite Strength Indices

Descriptive statistics and correlation analyses for the peak torque-based composite strength indices are presented in Table 2. At the unadjusted level, composite ankle strength indices showed moderate unadjusted associations with KBS. Dominant ASI showed a moderate unadjusted association with KBS (r = 0.48, *p* = 0.02), and non-dominant ASI showed a moderate unadjusted association with KBS (r = 0.42, *p* = 0.04). Dominant ASI normalized to body mass showed a moderate unadjusted association with KBS (r = 0.45, *p* = 0.02), while the non-dominant normalized ASI also showed a moderate unadjusted association with KBS (r = 0.39, *p* = 0.05). Dominant and non-dominant KSI values were 281.20 ± 64.69 (170–442) and 274.04 ± 78.87 (175–521), respectively. Following false discovery rate adjustment, none of the composite strength indices (ASI, KSI, PPSI, SPSI) remained statistically significant (all *p*(FDR) > 0.05).

## 4. Discussion

This study examined the relative associations between joint-level strength capacity and generalized neuromuscular activation and maximal soccer kicking ball speed under controlled conditions. The primary finding was that, following false discovery rate (FDR) correction, none of the mechanical or neuromuscular variables demonstrated a statistically significant association with maximal ball speed. These results indicate that, when evaluated outside a task-specific context, isolated isometric joint-level strength measures and generalized neuromuscular activation have limited explanatory value for inter-individual differences in maximal kicking performance. It is important to recognize that isometric strength represents a static measure that does not fully reflect the high-velocity and stretch–shortening cycle characteristics of soccer kicking. Accordingly, the absence of significant associations may partly reflect a biomechanical mismatch between the testing modality and the performance task. High-velocity isokinetic assessments may provide a more task-specific representation of kicking mechanics [11]; however, such approaches were beyond the scope of the present study, which aimed to isolate maximal force-generating capacity independent of movement velocity. This represents a reductionist approach that may not fully reflect the integrated and task-specific nature of soccer kicking performance.

From a statistical perspective, the disappearance of moderate associations after FDR correction highlights the importance of controlling for false positives when evaluating multiple interrelated predictors. These findings suggest that previously reported associations in similar contexts may partly reflect inflated Type I error rates when multiple comparisons are not adequately controlled. In this respect, the present study contributes a more conservative and methodologically robust evaluation of the relationship between strength-related variables and kicking performance. Nevertheless, in the unadjusted analyses, consistent moderate associations were observed at the ankle level for both individual torque variables (plantar flexion and dorsiflexion) and the composite ankle strength index (ASI), as well as for phase-oriented cross-joint composite indices integrating ankle and knee contributions. However, these findings should be interpreted as descriptive, as they did not retain statistical significance following FDR correction. In this context, combining agonist and antagonist torque components within a single index may reduce variance among interrelated variables and provide a more integrative representation of distal joint capacity.

From a biomechanical standpoint, the consistent directional trends observed in the unadjusted analyses may be interpreted within the framework of the kinetic chain model of soccer kicking. This model emphasizes that force production is not generated in isolation at a single joint but is transmitted through coordinated interactions from proximal to distal segments [1,4,7]. In this context, considering knee and ankle torque capacities together may provide a more holistic representation of proximal-to-distal force transfer, particularly during the propulsion and swing phases of the kicking motion. The ankle joint may play a functional role in the final stage of energy transfer by regulating foot orientation, modulating joint stiffness, and shaping impact mechanics at ball contact [1,9,33]. Moreover, the inclusion of both plantar flexion and dorsiflexion within composite ankle indices aligns with the role of antagonistic co-contraction in joint stabilization and stiffness regulation [34,35,36]. During the late swing and impact phases, plantar flexors contribute to resistance against deformation at ball contact, whereas dorsiflexors assist in controlling foot positioning [37,38]. The similar directional trends observed across dominant and non-dominant limbs may further suggest that distal joint capacity contributes bilaterally, consistent with the stabilizing role of the support leg in effective energy transfer [39,40]. However, as none of these associations remained statistically significant after FDR correction, all biomechanical interpretations should be considered exploratory and interpreted with caution.

Despite the well-established role of the knee joint in generating distal segment velocity during the swing phase, no significant association was found between isolated isometric knee extension strength and ball speed in the present study. This finding does not imply that the knee joint is unimportant for kicking performance. Rather, it suggests that maximal torque capacity assessed under static conditions may represent only a partial indicator of performance-related capacity in trained athletes. Previous strength profiling studies similarly indicate that static knee strength measures have limited discriminative value for functional soccer performance outcomes [41]. Evidence from kinematic and coordination-based analyses further suggests that the contribution of the knee joint to ball speed cannot be explained solely by torque magnitude, but is instead dependent on angular velocity generation, precise intersegmental timing, and phase-specific organization of the kicking sequence [40,42]. Accordingly, isolated proximal torque measures may not adequately reflect the neuromechanical strategies underlying maximal kicking performance. The phase-oriented cross-joint composite indices used in the present study were designed to approximate coordinated force production across joints; however, the current findings do not support definitive conclusions regarding their predictive value.

Generalized neuromuscular activation assessed during a standardized squat jump was not significantly associated with kicking ball speed. Although EMG recorded during a squat jump provides a reliable estimate of overall explosive neuromuscular output, it does not adequately capture the phase-specific activation patterns and intermuscular coordination characteristic of soccer kicking [18,19,20]. In high-skill ballistic actions, performance appears to depend less on the magnitude of activation and more on the temporal organization and mechanical integration of muscle activity within the task. Accordingly, neuromuscular activation assessed outside the kicking context may have limited capacity to explain inter-individual differences in maximal ball speed.

A deliberate trade-off was made in the study design between measurement reliability and ecological validity. Strength assessments were conducted under isometric conditions, and neuromuscular activation was evaluated using a standardized squat jump task. While these approaches provide controlled, reliable, and reproducible measurements, they do not fully capture the dynamic, task-specific coordination and impact-related characteristics of soccer kicking. Therefore, the present findings should be interpreted within the context of controlled laboratory-based assessments rather than as direct representations of match-specific performance.

Several limitations should be acknowledged. The cross-sectional design precludes causal interpretation. The absence of statistically significant associations following multiple comparison correction may also reflect limited sensitivity to detect small-to-moderate effects. In this context, the assumption of a large effect size in the a priori power analysis may have resulted in an underestimation of the required sample size, particularly for detecting smaller effects after correction. Furthermore, while composite strength indices provide an integrative representation of joint-level capacity, they may obscure the relative contributions of individual torque components, as both joint-specific (ASI, KSI) and phase-oriented cross-joint indices (PPSI, SPSI) aggregate torque across multiple joints and movement phases. Finally, sEMG recordings were limited to the dominant limb and did not include key proximal muscles involved in the swing phase of kicking. In particular, deep muscles such as the iliopsoas cannot be reliably assessed using surface EMG, further limiting the interpretation of proximal and bilateral neuromuscular contributions within the kinetic chain.

From an applied perspective, the absence of statistically significant associations after multiple comparison correction does not support the direct translation of these findings into performance-enhancing prescriptions. Rather, the results suggest that isolated isometric strength assessments and squat jump-based neuromuscular measures are not sufficient or reliable tools for predicting or evaluating maximal kicking performance. Although consistent directional trends were observed at the ankle level in unadjusted analyses, these should be considered exploratory and interpreted with caution.

The observed association between dominant knee flexion mean torque and ball speed suggests that knee flexors should not be entirely disregarded in performance evaluation. However, the current evidence is insufficient to justify isolated or prioritized interventions targeting knee flexors in training program design. Overall, lower-limb mechanical capacity should be considered as one component within a multifactorial performance structure that also includes technical, coordinative, and tactical determinants.

## 5. Conclusions

After correction for multiple comparisons, neither isolated isometric strength measures nor generalized neuromuscular activation assessed during a squat jump demonstrated statistically significant associations with maximal soccer kicking ball speed. These findings suggest that such isolated and non-task-specific assessments may have limited explanatory capacity for inter-individual differences in kicking performance. Taken together, these findings highlight the importance of dynamic, task-specific, and velocity-dependent factors in understanding soccer kicking performance. Future research should prioritize task-specific and velocity-dependent assessments to better capture the neuromechanical factors underlying maximal kicking performance.

## Figures and Tables

**Table 1:** Descriptive statistics of all mechanical and neuromuscular variables.

	Variables	Mean ± SD	Range	r	*p*	*p*(FDR)
	KBS (km·h^−1^)	92.28 ± 8.93	75–120			
Plantar Flexion	DPT	61.48 ± 27.93	18–125	0.38	0.06	0.133
	DPT (%BW)	78.88 ± 34.17	21–155	0.35	0.09	0.150
	NDPT	55.52 ± 26.79	24–118	0.36	0.07	0.133
	NDPT (%BW)	70.92 ± 31.66	27–125	0.36	0.07	0.133
	DAT	50.56 ± 21.74	16–96	0.41	0.04	0.133
	DAT (%BW)	65.16 ± 26.72	18–119	0.39	0.05	0.133
	NDAT	44.60 ± 21.22	20–88	0.24	0.24	0.343
	NDAT (%BW)	56.84 ± 25.37	21–107	0.23	0.27	0.360
Dorsiflexion	DPT	57.20 ± 12.95	41–87	0.47	0.02	0.100
	DPT (%BW)	73.00 ± 13.63	42–98	0.45	0.02	0.100
	NDPT	57.28 ± 11.23	39–87	0.41	0.04	0.133
	NDPT (%BW)	74.16 ± 14.74	30–95	0.27	0.20	0.333
	DAT	51.60 ± 12.12	38–80	0.39	0.05	0.133
	DAT (%BW)	66.36 ± 12.94	36–89	0.35	0.09	0.150
	NDAT	52.08 ± 10.39	34–79	0.40	0.05	0.133
	NDAT (%BW)	67.56 ± 14.11	27–92	0.26	0.20	0.333
Flexion	DPT	82.12 ± 21.88	47–118	0.37	0.07	0.133
	DPT (%BW)	104.00 ± 23.91	60–149	0.35	0.08	0.145
	NDPT	73.16 ± 19.42	47–117	0.28	0.17	0.283
	NDPT (%BW)	92.68 ± 20.33	60–131	0.24	0.25	0.357
	DAT	68.64 ± 23.49	14–107	0.52	0.01	0.100
	DAT (%BW)	90.04 ± 22.33	51–131	0.37	0.07	0.133
	NDAT	62.80 ± 17.89	39–100	0.25	0.24	0.343
	NDAT (%BW)	79.96 ± 18.78	48–116	0.18	0.39	0.520
Extension	DPT	199.08 ± 51.47	108–324	−0.01	0.96	0.990
	DPT (%BW)	255.64 ± 61.43	98–361	−0.09	0.68	0.872
	NDPT	200.88 ± 67.69	115–404	0.03	0.90	0.990
	NDPT (%BW)	257.44 ± 79.41	101–408	−0.07	0.74	0.949
	DAT	175.32 ± 46.55	94–279	−0.01	0.94	0.990
	DAT (%BW)	224.56 ± 54.40	89–322	−0.10	0.65	0.872
	NDAT	175.96 ± 61.94	104–370	0.08	0.69	0.872
	NDAT (%BW)	225.08 ± 69.51	86–361	0.00	1.00	1.000
sEMG (%MVIC)	RF	36.15 ± 17.83	15.55–100	−0.01	0.97	1.000
	SOL	39.09 ± 14.85	18.08–85.94	−0.04	0.86	0.956
	BF	63.88 ± 38.01	3.58–144.09	−0.32	0.12	0.240
	TA	26.20 ± 9.00	8.52–42.86	0.07	0.72	0.949
	GM	120.66 ± 90.26	30.72–375	0.10	0.64	0.872
	GL	108.46 ± 76.98	32.75–301.16	−0.08	0.70	0.949
	VL	36.32 ± 19.15	12.2–100.57	−0.15	0.48	0.686
	VM	34.73 ± 11.12	13.58–55.22	−0.10	0.62	0.872

Note: Values are presented as mean ± standard deviation (SD) and minimum–maximum range. Pearson correlation coefficients (r) indicate associations between each variable and kicking ball speed (KBS). Both unadjusted *p* values and false discovery rate-adjusted *p* values [*p*(FDR); Benjamini–Hochberg correction] are reported. Abbreviations: KBS, kicking ball speed; DPT, dominant peak torque; NDPT, non-dominant peak torque; DAT, dominant average torque; NDAT, non-dominant average torque; %BW, normalized to body mass; RF, rectus femoris; SOL, soleus; BF, biceps femoris; TA, tibialis anterior; GM, gastrocnemius medialis; GL, gastrocnemius lateralis; VL, vastus lateralis; VM, vastus medialis; sEMG, surface electromyography; %MVIC, percentage of maximal voluntary isometric contraction.

**Table 2:** Descriptive statistics of composite strength indices and their correlations with kicking ball speed.

	Variables	Mean ± SD	Range	r	*p*	*p*(FDR)
KSI	DPT	281.20 ± 64.69	170–442	0.12	0.580	0.844
	DPT (%BW)	359.64 ± 74.71	158–489	0.04	0.840	0.896
	NDPT	274.04 ± 78.87	175–521	0.09	0.660	0.844
	NDPT (%BW)	350.12 ± 89.98	164–539	−0.01	0.980	0.980
ASI	DPT	118.68 ± 34.60	59–181	0.48	0.020	0.080
	DPT (%BW)	151.88 ± 40.19	63–221	0.45	0.020	0.080
	NDPT	112.80 ± 33.77	70–205	0.42	0.040	0.091
	NDPT (%BW)	145.08 ± 38.73	57–220	0.40	0.050	0.114
PPSI	DPT	260.56 ± 67.44	135–422	0.15	0.482	0.771
	DPT (%BW)	334.52 ± 77.51	119–453	0.09	0.680	0.844
	NDPT	256.40 ± 82.42	154–522	0.14	0.504	0.771
	NDPT (%BW)	328.36 ± 91.97	128–506	0.07	0.755	0.863
SPSI	DPT	139.32 ± 30.03	104–204	0.47	0.017	0.080
	DPT (%BW)	177.00 ± 30.12	102–235	0.48	0.014	0.080
	NDPT	130.44 ± 25.50	94–204	0.43	0.033	0.091
	NDPT (%BW)	166.84 ± 27.9	93–214	0.32	0.125	0.200

Note: Values are presented as mean ± standard deviation (SD) and minimum–maximum range. Pearson correlation coefficients (r) indicate associations between each variable and kicking ball speed (KBS). Both unadjusted *p* values and false discovery rate-adjusted *p* values [*p*(FDR); Benjamini–Hochberg correction] are reported. Abbreviations: DPT, dominant peak torque; NDPT, non-dominant peak torque; %BW, normalized to body mass; KSI, Knee Strength Index; ASI, Ankle Strength Index; PPSI, Push-Phase Strength Index; SPSI, Swing-Phase Strength Index.

## Data Availability

The data presented in this study are available from the corresponding authors upon reasonable request due to ethical and privacy restrictions.
